# β-Carotene Increases Activity of Cytochrome P450 2E1 during Ethanol Consumption

**DOI:** 10.3390/antiox11051033

**Published:** 2022-05-23

**Authors:** Cristian Sandoval, Luciana Mella, Karina Godoy, Khosrow Adeli, Jorge Farías

**Affiliations:** 1 Escuela de Tecnología Médica, Facultad de Salud, Universidad Santo Tomás, Los Carreras 753, Osorno 5310431, Chile; 2 Departamento de Ingeniería Química, Facultad de Ingeniería y Ciencias, Universidad de La Frontera, Temuco 4811230, Chile; 3 Departamento de Ciencias Preclínicas, Facultad de Medicina, Universidad de La Frontera, Temuco 4811230, Chile; 4 Carrera de Tecnología Médica, Facultad de Medicina, Universidad de La Frontera, Temuco 4811230, Chile; l.mella01@ufromail.cl; 5 Núcleo Científico y Tecnológico en Biorecursos (BIOREN), Universidad de La Frontera, Temuco 4811230, Chile; karina.godoy@ufrontera.cl; 6 Molecular Medicine, Research Institute The Hospital for Sick Children University of Toronto, Toronto, ON M5G 1X8, Canada; khosrow.adeli@sickkids.ca

**Keywords:** alcohol intake, alcoholic fatty liver disease, antioxidant treatment, chronic alcohol consumption

## Abstract

One of the key routes through which ethanol induces oxidative stress appears to be the activation of cytochrome P450 2E1 at different levels of ethanol intake. Our aim was to determine if oral β-carotene intake had an antioxidant effect on *CYP2E1* gene expression in mice that had previously consumed ethanol. C57BL/6 mice were used and distributed into: control (C), low-dose alcohol (LA), moderate-dose alcohol (MA), β-carotene (B), low-dose alcohol+β-carotene (LA + B), and moderate-dose alcohol+β-carotene (MA + B). Animals were euthanized at the end of the experiment, and liver tissue was taken from each one. *CYP2E1* was measured using qPCR to detect liver damage. The relative expression level of each RNA was estimated using the comparative threshold cycle (Ct) technique (2^−ΔΔCT^ method) by averaging the Ct values from three replicates. The LA+B (2267 ± 0.707) and MA+B (2.307 ± 0.384) groups had the highest *CYP2E1* fold change values. On the other hand, the C (1.053 ± 0.292) and LA (1.240 ± 0.163) groups had the lowest levels. These results suggest that ethanol feeding produced a fold increase in *CYP2E1* protein in mice as compared to the control group. Increased *CYP2E1* activity was found to support the hypothesis that β-carotene might be dangerous during ethanol exposure in animal models. Our findings imply that β-carotene can increase the hepatic damage caused by low and high doses of alcohol. Therefore, the quantity of alcohol ingested, the exposure period, the regulatory mechanisms of alcoholic liver damage, and the signaling pathways involved in the consumption of both alcohol and antioxidant must all be considered.

## 1. Introduction

Excessive alcohol drinking has been linked to several deadly illnesses, including cancer, cirrhosis of the liver, vascular disease, neuropsychiatric illness, as well as diabetes [1,2,3]. In addition, that increased oxidative stress can produce hepatic damage in people has been demonstrated [4,5].

Alcoholic fatty liver, alcoholic hepatitis, and cirrhosis are all caused by ethanol metabolism [6,7]. In the hepatic metabolization, enzymes such *CYP450 2E1* (*CYP2E1*), alcohol dehydrogenase (ADH), and catalase (CAT) are involved in the oxidative pathway, whereas through the non-oxidative pathway the fatty acid ethyl ester (FAEE) synthase creates FAEEs [8,9].

The microsomal respiratory chain and *CYP2E1*-dependent microsomal monooxygenase system are the main sources of ROS during alcohol intake. As to its ability to produce a diversity of hepatotoxic substrates, such as N-nitrosodimethylamine, alcohol, acetaminophen, and carbon tetrachloride, *CYP2E1* is of particular interest [10]. According to this theory, alcohol-induced activation of *CYP2E1* is one of the primary mechanisms by which alcohol produces oxidative stress. Furthermore, *CYP2E1* oxidizes ethanol to form a very reactive particle that might contribute to alcohol’s harmfulness, acetaldehyde [11].

The primary contributor to the development of alcohol-mediated liver damage, extracellular matrix changes, and inflammation has been identified as acetaldehyde [12,13]. The generation of reactive oxygen species (ROS) and a redox potential imbalance (NAD/NADH) generate its effects. It also links to DNA, producing oncogenic chemicals like 1,N^2^-(3-hydroxypropane)-2′-deoxyguanosine, and creates protein aggregates in hepatocytes, restricting protein synthesis and promoting hepatomegaly. It also forms salsolinol when it reacts with dopamine, which can contribute to alcohol dependency [14,15]. 

Alcohol-mediated oxidative stress and toxicity have previously been examined in animal models and in vitro studies [16,17]. In consequence, these results have sparked fresh research into new pathophysiological targets that may be used to treat alcoholic liver disease (ALD). In effect, enzymatic mechanisms such as catalase, superoxide dismutase, and glutathione peroxidase and reductase, as well as non-enzymatic mechanisms, might be used to block the hepatocyte’s antioxidant defense [18,19,20]. Many antioxidants, including silymarin, N-acetylcysteine, vitamin E, and S-adenosylmethionine (SAMe), have been examined in recent clinical investigations, although the results have been inconsistent [18,19,21,22]. Therefore, this study aimed to examine the consequences of β-carotene supplementation on *CYP2E1* activity in C57BL/6 mice exposed to alcohol consumption.

## 2. Materials and Methods

### 2.1. Animals

Thirty male C57BL/6 mice were used (*Mus musculus*), 50 days old, from the Chilean Public Health Institute. They were kept for 30 days under standardized conditions and a 12 h light/dark cycle (08:00 a.m.–08:00 p.m./08:00 p.m–8:00 a.m.), with a standard laboratory diet (AIN-93M) and water *ad libitum* to help them adjust to their new environment in the Animal Facility of the Center of Excellence in Morphological and Surgical Studies (CEMyQ) at the Universidad de La Frontera. The animals were handled according to the recommendations published by the Institute for Laboratory Animal Research [23]. The Scientific Ethics Committee of the Universidad de La Frontera has approved this project (Nº051/2020).

The mice were split into six groups on the first day of the experiment (day 1): 1. control (C); 2. low-dose alcohol (LA): low-dose alcohol consumption (3% *v/v ad libitum*) for 28 days [24]; 3. moderate-dose alcohol (MA): moderate-dose alcohol consumption (7% *v/v ad libitum*) for 28 days [24]; 4. β-carotene (B): administration of 0.52 mg/kg body weight/day of β-carotene for 28 days [25]; 5. low-dose alcohol + β-carotene (LA + B): low-dose alcohol consumption plus administration of 0.52 mg/kg body weight/day of β-carotene for 28 days; and 6. moderate-dose alcohol + β-carotene (MA + B): moderate-dose alcohol consumption plus administration of 0.52 mg/kg body weight/day of β-carotene for 28 days.

Chronic ethanol administration was given according to the modified liquid diet of Lieber-DeCarli [24,26]. β-carotene was orally administered at a dose of 0.52 mg/kg body weight/day [25].

### 2.2. Euthanasia

On day 28, at the end of the experiment, the animals were fasted for 6 h and euthanized with sodium pentobarbital.

### 2.3. Liver Tissue

Each animal’s liver tissue (*n* = 30) was obtained after euthanasia. Liver samples were extracted as soon as possible and placed in autoclaved microtubes containing lysis solution (RNeasy Mini Kit, QIAGEN) for RNA stabilization. They were then stored at room temperature for 30 min. The samples were then frozen in liquid nitrogen before being carried to the freezer room. The frozen samples were then transferred to an ultra-freezer and stored at −80 °C until they were utilized.

### 2.4. Extraction of RNA and cDNA Synthesis

The liver sample was crushed into a fine powder in liquid nitrogen with a prechilled mortar and pestle, then combined with the TRIzol (QIAGEN Diagnostics GmbH, Germany)/lysis buffer given with the kits and extracted according to the methodology previously described [27]. Using High-Capacity cDNA Reverse Transcription Kits, the mRNA strand was reverse transcribed into single-stranded cDNA (Applied Biosystems, Waltham, MA, USA). The cDNA was subsequently amplified using TaqMan^TM^ Universal PCR Master Mix in a quantitative PCR (qPCR) (Applied Biosystems, Waltham, MA, USA).

### 2.5. Quantification of RNA from Liver Tissue

The amount and integrity of the isolated total RNA were analyzed using the Qubit^TM^ 4.0 Fluorometer (Life Technologies, Thermo Fisher Scientific Inc., Waltham, MA, USA). The RNA IQ# was estimated from the fraction of large and small RNA in the sample. The RNA IQ# is a number that ranges from 1 to 10, where a high number suggests that most of the RNA in the sample is large and/or organized. On the other hand, a low IQ# indicates that the sample is largely small RNA with little tertiary structure. The manufacturer’s instructions were followed while using the standard Qubit^TM^ RNA HS Assay Kit (Life Technologies, Thermo Fisher Scientific Inc.). The Qubit^TM^’s functioning solution was developed in accordance with the manufacturer’s standards. We added to each assay tube 180 μL of working solution, up to 20 μL of RNA, and enough water to make the final volume 200 μL. For the standard tubes, 10 μL of Qubit^TM^ RNA Standard solutions were placed into the tubes. The assay tubes were vortexed for 2–3 s, centrifuged for 5 s, and then left at room temperature for 2 min before being measured with the Qubit^TM^ Fluorometer. For the Qubit™ Assay, RNA sample concentration was calculated as: [Concentration of your sample] = QF value (the value given by the Qubit^®^ 4.0 Fluorometer) × (200/the number of microliters of sample put into the assay tube). Three different measurements were taken on each sample.

### 2.6. Quantitative Real-Time PCR

The expression levels of *CYP2E1*, family 2, subfamily e, polypeptide 1, β-actin (*ACTB*), and glyceraldehyde-3-phosphate dehydrogenase (*GAPDH*) were analyzed using qPCR. The data were normalized according to the mRNA expression levels of housekeeping genes, such as *ACTB* and *GAPDH*. *CYP2E1* was the target gene. All qPCR experiments were performed using the QuantStudio3 system (Applied Biosystems, Waltham, MA, USA). All the amplifications were done using TaqMan^TM^ Universal PCR Master Mix (Applied Biosystems, Waltham, MA, USA). The TaqMan gene expression test is a ready-to-use 5’-3’ Taq polymerase assay containing TaqMan^®^ dye-labeled probes (FAM/MGB) and desired primers, as presented in Table 1. In addition, housekeeping genes are presented in Table 2.

After a 10-min denaturation phase at 95 °C, 40 cycles at 95 °C for 30 s, 60 °C for 30 s, and 72 °C for 30 s were performed. A melting curve analysis of each qPCR was carried out after each cycle. The number of times the reporter dye in the PCR reaction crossed a software-defined threshold, which was computed automatically by the QuantStudio^TM^ Design & Analysis Software, is referred to as the ‘Ct,’ or threshold cycle (version 1.3, Applied Biosystems, Waltham, MA, USA). The relative expression level of each RNA was estimated using the comparative threshold cycle (Ct) technique (2^−ΔΔCt^ method) by averaging the Ct values from three replicates. We utilized the threshold cycle values automatically generated by the qPCR equipment for the 2^−ΔΔCt^ technique.

### 2.7. Statistical Analysis

The Kolmogorov–Smirnov test (data normality analysis) and Levene’s test were used to assess differences in quantitative data (homoscedasticity of the variances). One-way ANOVA was used to analyze the differences among the groups. Tukey’s HSD test or Dunnett’s T3 test were used to realize such a post-hoc test, as applicable. *p* < 0.05 was considered statistically significant (GraphPad Prism 6, GraphPad Software Inc., San Diego, CA, USA).

## 3. Results

### 3.1. Statistical Analysis

RNA was extracted from liver samples and was analyzed for integrity and quality. The assay kit was prepared to be precise concerning initial RNA sample concentrations of 0.5 to 1200 ng/L, yielding a detection range of 10 to 1200 ng, depending on sample volume. A total of 18 specimens had sufficient yield (RNA IQ# >8) to proceed to amplification through a quantitative PCR (qPCR) using TaqMan^TM^ Universal PCR Master Mix (Applied Biosystems, CA, USA). It has been described that RNA is pure and satisfactory for downstream studies if RIN > 7 [28].

### 3.2. Quantitative Real-Time PCR

In gene quantification analysis, data normalization in qPCR is a critical step [29,30]. Indeed, depending on the experimental settings and pathophysiology of the examined tissue, mRNA levels of the required housekeeping genes, *ACTB* and *GAPDH*, are likely to change to the point where normalization becomes erroneous and/or deceptive.

**Figure 1:**
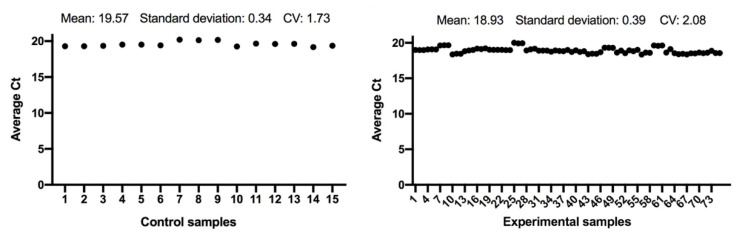
Comparison of *ACTB* gene expression from control and experimental groups, respectively.

**Figure 2:**
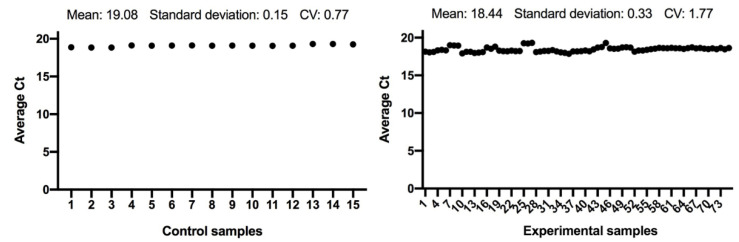
Comparison of *GAPDH* gene expression from control and experimental groups, respectively.

The expression of the *ACTB* and *GAPDH* genes changes very little between the control and the experimental samples, as seen in Figure 1 and Figure 2; and has a low variability of expression. As a result, the internal control genes *ACTB* and *GAPDH* may be used to provide accurate and consistent findings. *ACTB* and *GAPDH* resolve differences in templates starting with the amount and operational loading errors [31].

The reference RNA utilized in the standard curve approach is highly efficient and stable. Moreover, this RNA has been useful to determine absolute comparative quantification of target genes by qPCR. 

**Figure 3:**
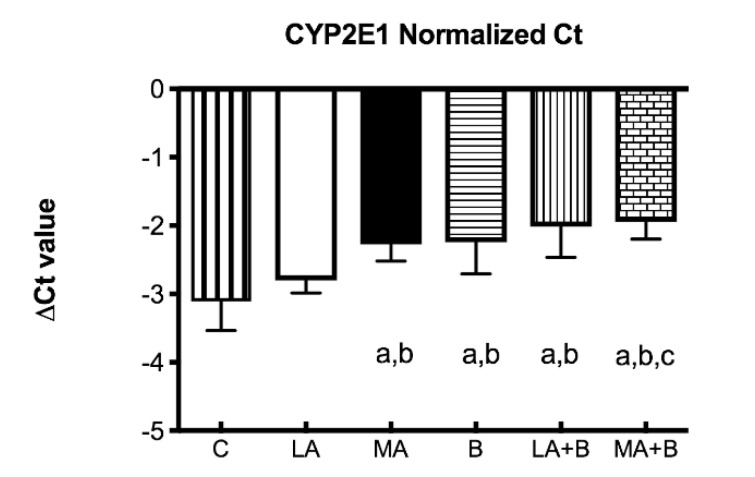
Delta Ct values of *CYP2E1* mRNA of control and experimental groups. Bars represent mean ± SD values of ∆Ct per group; a: significant differences (*p* < 0.05) with the C group; b: significant differences (*p* < 0.05) with the LA group; c: significant differences (*p* < 0.05) with the MA group.

Since chronic ethanol feeding elevates *CYP450 2E1*, the ΔCT values were determined for *CYP2E1* mRNA expression profiles of control and experimental groups after 28 days of ethanol and/or ß-carotene administration (Figure 3). The ΔCT value is the gap among the target gene and housekeeping genes, described as:

∆CT = average CT (a target gene) − average CT (housekeeping genes)(1)

The 2^−ΔΔCT^ comparative approach was used to estimate relative gene expression. All data were regulated to *ACTB* and *GAPDH* mRNA content. Comparative RNA expression study in experimental versus control groups (calibrator) was performed as follows:

Experimental groups: ΔCt = Ct (target) − Ct (housekeeping genes) Control group: ΔCt = Ct (target) − Ct (housekeeping genes) ΔΔCt = ΔCt (experimental groups) − ΔCt (control group) Ratio = 2^−ΔΔCt^ (2)

The average ΔCt value of housekeeping gene RNA was subtracted from the average Ct value of the control and experimental groups, yielding the Ct value. The ΔΔCt value was obtained by subtracting the control group’s ΔCt value from the experimental groups’ ΔCt value [32]. The ratio 2^−ΔΔCt^ was used to determine the fold of enrichment values.

**Figure 4:**
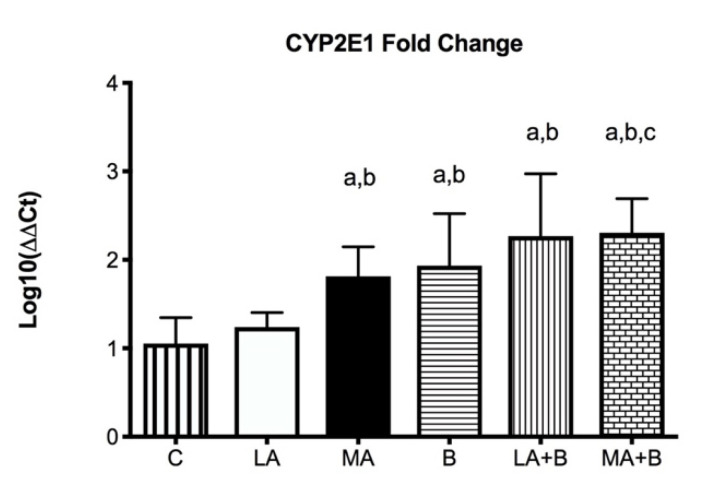
*CYP2E1* mRNA fold change is expressed as fold change using the ΔΔCt method in experimental groups with respect to the control group (calibrator). Bars represent mean ± SD values of fold change per group; a: significant differences (*p* < 0.05) with the C group; b: significant differences (*p* < 0.05) with the LA group; c: significant differences (*p* < 0.05) with the MA group.

Figure 4 displays that following 28 days of alcohol intake, levels of *CYP2E1* were bigger 2267 ± 0.707-fold in LA+B and 2.307 ± 0.384-fold in MA+B groups after ethanol and β-carotene exposure. No significant differences were found between C and LA groups. 

The housekeeping genes allow the target gene’s gene expression pattern to be normalized against the quantity of input RNA or cDNA. They were adjusted for probable RNA degradation, sample management variations, reverse-transcription efficacy differences, the existence of inhibitors in the RNA sample and RNA content, and sample handling differences. The comparative approach (ratio 2^−ΔΔCT^) was applied, with *ACTB* and *GAPDH* serving as housekeeping genes and the control group serving as a calibrator [33].

## 4. Discussion

### 4.1. Summary of Key Findings and Interpretation

For reference samples, the relative gene expression is commonly adjusted to 1 since CT equals 0 and hence 20 equals 1. The 2^−ΔΔCT^ method approach assumes that all samples have a consistent PCR amplification efficiency of 100 percent [33,34]. The number 2 is 1 plus the PCR amplification efficiency (100 %). This assumption simplifies the method and ensures that it is valid in ideal circumstances. However, since there are variables such as the existence of PCR inhibitors or enhancers, extraction of RNA, and various primers, probes, and enzymes, PCR efficiency cannot be guaranteed.

Ethanol dependence is a disease that progresses nearly five years after the primary alcohol use starts and it takes nearly 15–20 years for the alcohol addict to request medical care [35,36]. While much of the effort on alcohol metabolism has been on ADH, chronic alcohol consumption might raise levels of other enzymes such as *CYP2E1* [15,30,37,38,39]. In effect, elevated levels of *CYP2E1* in the liver of patients with alcoholic and nonalcoholic liver diseases have been shown [40]. As expected, ethanol has shown that feeding mice using the Lieber-DeCarli liquid diet results in a fold increase in *CYP2E1* levels compared to control (Figure 3 and Figure 4). Under these conditions, previous studies using the same protocol have shown alcohol feeding produces fatty liver and raises LDL-C levels [39].

Following alcohol intake, the microsomal monooxygenase system, and the microsomal respiratory chain, both of which rely on *CYP2E1*, are the major generators of ROS in hepatocytes. Because of its propensity to process and stimulate diverse hepatotoxic substrates in the liver, cytochrome P450 2E1 is particularly essential in the prevention of carbon tetrachloride, alcohol, N-nitroso dimethylamine, and acetaminophen turning into more toxic compounds [10]. The activation of *CYP2E1* by ethanol seems to be one of the keyways by which alcohol produces oxidative stress. In effect, *CYP2E1* also converts ethanol to acetaldehyde, a highly reactive molecule that contributes to ethanol toxicity [11]. Furthermore, heavy alcohol use appears to be associated with *CYP2E1* blood expression [41]. Due to enhanced NADPH oxidase activity and strong production of O_2_ and H_2_O_2_ radicals even in the absence of substrate, *CYP2E1* is a potent ROS generator [42].

Previous research has established the alcohol and β-carotene dosages, as well as the treatment times [24,25]. In this regard, low doses of oral β-carotene supplementation have been shown to protect against liver damage caused by the antioxidant pathway [20,43]. Our findings have reported greater levels of *CYP2E1* fold-change in the MA, LA+B, and MA+B groups in comparison to the C group and even the LA group (Figure 4, *p* < 0.05). The C and LA groups had the lowest levels (1.053 ± 0.292 and 1.240 ± 0.163, respectively). Hence, our findings show that ß-carotene increases the activity of *CYP2E1* during ethanol consumption in low and moderate doses. Conversely, previous studies using the same protocol found it could prevent alcoholic liver disease and improve health when biochemical markers and histopathological and transmission electron microscopy are used in the evaluation [20,39]. In effect, reduced oxidative stress and reduced *CYP2E1* activity have been found to support the protection provided by induced-β-carotene in a moderate alcohol intake. These discrepancies demonstrate the need to identify the action pathway of β-carotene during ethanol metabolism, which could be directly related to acetaldehyde and even acetate. Despite these findings, it is necessary to understand that these discrepancies with other studies are mainly due to the amount of alcohol drunk, the exposure period, the regulatory mechanisms of alcoholic liver damage, and the signaling pathways involved in the consumption of both alcohol and antioxidants. In fact, previous reviews have described these discrepancies using vitamins and supplements against alcoholic liver disease [44].

Although increases in *CYP2E1* mRNA can occur at very high blood ethanol levels [45], ethanol induction of *CYP2E1* is mostly posttranscriptional, suggesting the stability of *CYP2E1* against proteosome-mediated destruction. Ethanol is both a ligand and a substrate for *CYP2E1*, which explains its ability to stabilize and prolong the half-life of the enzyme [46,47]. This study also shown that combining ethanol with p-carotene causes a more severe hepatic damage in C57BL/6 mice than either drug alone, raising concerns about the use of B-carotene as a source of retinol and as an anticancer agent when substantial alcohol use or abuse is present. It has been frequently suggested that a lack of carotenoids in a diet is related to an increased risk of cancer [48,49,50], while several studies have failed to show such a link [51,52].

### 4.2. Scope and Limitations

The aim of this research was to evaluate the consequences of β-carotene on *CYP2E1* activity of C57BL/6 mice exposed to alcohol exposure. In addition, our data update the existing linkage between *CYP2E1* and β-carotene therapy. However, the absence of information linking alcohol dehydrogenase and aldehyde dehydrogenase expressions with antioxidant treatments remains a limitation of this research and must be attended to in future studies. Unfortunately, previous research results provide little support for this notion. Considering these data, seems that β-carotene exposure did not improve the hepatotoxic damage induced by alcohol exposure in C57BL/6 mice.

## 5. Conclusions

Because *CYP2E1* activity increases after moderate alcohol consumption and β-carotene, our findings imply that antioxidant therapies might be dangerous during ethanol exposure in animal models. Despite all the progress made in understanding the effects of antioxidant supplementation, future studies should use specific cell lines and clinical trials to better understand the relationship between alcohol consumption, antioxidant therapies, the signaling pathways involved, and enzymatic and non-enzymatic mechanisms.

## Figures and Tables

**Table 1:** Primers for gene targeting.

Gene	Gene Symbol	Assay	Chromosome Location	Amplicon Length
cytochrome P450, family 2, subfamily e, polypeptide 1	*Cyp2e1*	Mm00491127_m1	Chr.7: 140763832–140774981	83 bp

**Table 2:** Housekeeping genes for quantitative PCR.

Gene	Gene Symbol	Assay	Chromosome Location	Amplicon-Length
actx, E430023M04Rik, beta-actin	*Actb*	Mm00607939_s1	Chr.5: 142903116–142906724	115 bp
glyceraldehyde-3-phosphate dehydrogenase	*Gapdh*	Mm99999915_g1	Chr.6: 125161338–125166511	107 bp

## Data Availability

The data that support the findings of this study are openly available in “figshare” at https://doi.org/10.6084/m9.figshare.19766218.v1, accessed on 4 April 2022.
